# Do CTA measurements of annular diameter, perimeter and area result in different TAVI prosthesis sizes?

**DOI:** 10.1007/s10554-018-1394-1

**Published:** 2018-06-16

**Authors:** Barbora Horehledova, Casper Mihl, Babs M. F. Hendriks, Nienke G. Eijsvoogel, Jindrich Vainer, Leo F. Veenstra, Joachim E. Wildberger, Marco Das

**Affiliations:** 1 Department of Radiology and Nuclear Medicine, Maastricht University Medical Center, P.O. Box 5800, 6202 AZ Maastricht, The Netherlands; 2 CARIM School for Cardiovascular Diseases, Maastricht University Medical Center, Maastricht, The Netherlands; 3 Department of Cardiology, Maastricht University Medical Center, Maastricht, The Netherlands; 4 Department of Diagnostic and Interventional Radiology, Helios Kliniken Duisburg GmbH, Duisburg, Germany

**Keywords:** Aortic valve stenosis, Heart valve prosthesis implantation, Transcatheter aortic valve replacement, Multidetector computed tomography

## Abstract

Incorrect prosthesis size has direct impact on patient outcome after transcatheter aortic valve implantation (TAVI) procedure. Currently, annular diameter, area or perimeter may be used for prosthesis size selection. The aim was to evaluate whether the use different annular dimensions would result in the selection of different prosthesis sizes, when assessed in the same TAVI-candidate during the same phase of a cardiac cycle. Fifty consecutive TAVI-candidates underwent retrospectively ECG-gated computed tomography angiography (CTA). Aortic root dimensions were assessed in the 20% phase of the R–R interval. Annular short diameter, perimeter and area were used to select the prosthesis size, based on the industry recommendations for a self-expandable (Medtronic CoreValve; MCV) and balloon-expandable (Edwards Sapien XT Valve; ESV) valve. Complete agreement on selected prosthesis size amongst all three annular dimensions was observed in 62% (31/50; ESV) and 30% (15/50; MCV). Short aortic annulus measurement resulted in a smaller prosthesis size in 20% (10/50; ESV) and in 60% of cases (30/50; MCV) compared to the size suggested by both annular perimeter and area. In 18% (9/50; ESV) and 10% of cases (5/50; MCV) a larger prosthesis would have been selected based on annular perimeter compared to annular diameter and area. Prosthesis size derived from area was always in agreement with at least one other parameter in all cases. Aortic annulus area appears to be the most robust parameter for TAVI-prosthesis size selection, regardless of the specific prosthesis size. Short aortic annulus diameter may underestimate the prosthesis size, while use of annular perimeter may lead to size overestimation in some cases.

## Introduction

Transcatheter aortic valve implantation (TAVI) is a minimally invasive and alternative treatment of severe symptomatic aortic stenosis, originally only indicated in patients with high surgical risk, who are not suitable for an open-heart surgical aortic valve replacement [1–3]. However, with the publication of new 2017 ESC/EACTS Guidelines for the management of valvular heart disease, the indications for TAVI have expanded, because there is new evidence for TAVI also in the intermediate risk population [4]. Imaging plays a key role in pre-procedural planning, reliable selection of TAVI prosthesis and choice of suitable valve size [2, 3, 5, 6]. Precise pre-procedural imaging is therefore crucial to assure optimal patient outcome [2, 3, 5].

Early industry recommendations for transcatheter aortic valve size selection have been based on the annular diameter, which was assessed as the left ventricular outflow tract (LVOT) diameter in echocardiography [6]. Multidetector row computed tomography (MDCT), with the possibility of three-dimensional visualization, has proven to be superior in aortic root assessment and prediction of patient outcome [5, 7]. MDCT has therefore become the method of choice in TAVI planning [5, 8]. Implementation of routine three-dimensional evaluation of the aortic annulus allowed the manufacturers to extend the prosthesis sizing guidelines to three annular dimensions, namely annular diameter, area and perimeter [6, 9].

However, no further directions for their individual application or explanation of their associated relationship have been propounded in the industry guidelines. This has led to a flexible interpretation of industry guidelines. Annular area and perimeter have served either directly for prosthesis size selection or for further calculation of effective diameters. Therefore, up to five annular diameters (short, long, mean, area and perimeter derived effective diameters) have been frequently referred to in the literature as optional dimensions for TAVI planning [10–13]. Industry recommended diameter sizing thresholds have been applied to all five above-mentioned diameters without any adaptation, even though the systematic difference, described as increasing diameter length respectively from the short to effective and long diameter, has been regularly reported, possibly suggesting various prosthesis sizes [10, 11, 13].

To restrict from modification of industry recommendations in this study, we derive the TAVI prosthesis size directly from the measurement of annular area or perimeter, rather than from calculated effective diameters. For annular diameter, we evaluate the MDCT assessed short annular diameter, because it resembles the LVOT diameter assessed on the parasternal long axis view (PLAX) in echocardiography [6, 10] and therefore corresponds the best with the diameter sizing thresholds in industry guidelines.

To the best of our knowledge this is the first study to evaluate, whether the three aortic annulus dimensions stated in the industry guidelines can indeed be used interchangeably for TAVI planning. To this aim, we have assessed the aortic annulus short diameter, area and perimeter in the same phase of the cardiac cycle in fifty consecutive TAVI-candidates undergoing MDCT and recorded the suggested prosthesis size for each annular measurement.

## Materials and methods

### Patient population

Fifty consecutive TAVI candidates with severe and symptomatic aortic valve stenosis, who underwent pre-TAVI MDCT assessment of the aortic root, were retrospectively included in this study. Ethical approval and a waiver of informed consent were given by the local medical ethical research committee (METC reference number: 2017-0265).

### MDCT scan and contrast media protocol

A uniform dedicated scan protocol on a 2nd generation dual source MDCT (SOMATOM Definition Flash, Siemens, Forchheim, Germany), consisting of a low-pitch retrospective ECG-gated helical scan of the aortic root, followed by a high-pitch non ECG-triggered computed tomographic angiography (CTA) of the whole aorta, was used. The acquisition protocol is summarized in Table 1. The total of 120 ml of pre-warmed, monomeric, non-ionic, low osmolar iodinated contrast media (CM; Iopromide, Ultravist 300, Bayer, Berlin, Germany) was injected through an 18G needle in the antecubital vein following previously published tri-phasic CM injection protocol [8].

**Table 1:** Scan and contrast media protocols

Scan type	Retrospective ECG-gated	Non ECG-triggered
Scan direction	Cranio-caudal	
Tube voltage (kv)	100	
Quality ref. tube current (mAs)	320	150
Dose modulation	CARE Dose4D	
Rotation time (s)	0.28	
Pitch	0.17	3
Slice collimation	2 × 2 × 64 × 0.6	
Slice width (mm)	0.75/0.7	1.5/1.0
Reconstruction kernel	B26f	I30f
Contrast media	Iopromide 300 (Ultravist)	
Test bolus	20 ml CM × 7.2 ml/s followed by 15 ml NaCl × 7.2 ml/s	
Main bolus	75 ml CM × 7.2 ml/s (100%) 50 ml CM/NaCl × 7.2 ml/s (50/50%) 25 ml NaCl × 7.2 ml/s	
Iodine delivery rate (gI/s)	2.16	
Total iodine load (gI)	36	

*CM* contrast medium, *ECG* electrocardiogram, *gI* grams of iodine, *kV* kilovolt, *mAs* milliamper-second, *mGy* milligray, *ml* milliliter, *mm* millimeter, *NaCl* saline, *s* second

### Image reconstruction

Images were reconstructed with dedicated post processing software (SyngoVia™, version VB10A Siemens, Forchheim, Germany) with 0.75 mm slice thickness and 0.7 mm increment using a raw-data based iterative reconstruction (IR) algorithm (SAFIRE, Siemens Healthcare, Forchheim, Germany) with B26f kernel, strength 3. Images from the low-pitch retrospectively ECG-gated scan were reconstructed at 10 time points throughout the R-R interval at 10% increments (0–100% phase).

### Assessment of aortic annulus dimensions

The aortic annulus dimensions were assessed using multiplanar reformations (MPR) view on the oblique transversal plane, positioned at the level of the aortic annulus in such a way that the most basal portions of the aortic leaflets were equally distributed in the transversal plane view, in accordance with the expert consensus guidelines of the Society of Cardiovascular Computed Tomography (SCCT) [6]. This position allowed measurements of the short and long annular diameter as well as of the perimeter and area of the aortic annulus. To perform the measurements the 20% phase of the R–R interval (endsystole) was selected on the retrospectively ECG-gated low-pitch helical scans, according to our institutional standard [8].

The mean diameter was defined as an average of short and long annular diameter. The effective diameters derived from area (D_A_) and perimeter (D_P_) were calculated with commonly used and previously published formulas (Table 2) [8]. Annular eccentricity was quantified with the following equation:

**Table 2:** Formulas used for calculation of effective diameters and MCV sizing thresholds for annular area and diameter

	Formulas used for calculation of effective diameters	Formulas used for calculation of MCV sizing thresholds
Cross sectional area	${\text{}}{\text{}}{{\text{D}}_{{\text{A}}}}=2 \times \sqrt {\left( {\frac{{{\text{cross-sectional~area}}}}{\pi }} \right)}$	${\text{Cross-sectional~area}}~=~\frac{{\pi ~ \times ~{\text{diameter}}{^2}}}{4}$
Perimeter	${\text{}}{\text{}}{{\text{D}}_{{\text{P}}}}=\frac{{{\text{perimeter}}}}{{{{\uppi}}}}$	${\text{Perimeter}}=~\pi ~ \times ~{\text{diameter}}$

*D*_*A*_ effective diameter derived from annular area, *D*_*P*_ effective diameter derived from annular perimeter

$${\text{Annular~eccentricity}}~=\sqrt {\left[ {1 - {{\left( {\frac{{{\text{short~annular~diameter}}}}{{{\text{long~annular~diameter}}}}} \right)}^2}} \right]}$$

An eccentricity greater than 0 describes an elliptical aortic annulus, while an eccentricity of 0 describes a perfect circle [2].

### Transcatheter aortic valve prosthesis sizing

Transcatheter aortic valve sizes were theoretically selected based on the thresholds for the various dimensions of the aortic annulus as stated in the industry recommendations for balloon expandable (Edwards SapienValve XT; [ESV]; Edwards Lifesciences Corp, Irvine, USA) and self-expandable valve (Medtronic CoreValve; [MCV]; Medtronic, Minneapolis, USA) [6, 14, 15]. Recommendations are summarized in Table 3. The agreement between suggested valve sizes derived from individual annular dimensions was analyzed.

**Table 3:** Industry recomendations for transcatheter aortic valve prosthesis sizing

Edwards Sapien XT				
Valve size (= device diameter)	20 mm	23 mm	26 mm	29 mm
	Suitable annular dimensions			
Aortic annulus diameter [mm]	17–19	18–22	21–25	24–27
Aortic annulus area (mm^2^)	280–320	310–410	410–520	520–650
Aortic annulus perimeter (mm)	54–68	62–72	72–81	81–90

Medtronic CoreValve				
Valve size (= device diameter)	23 mm	26 mm	29 mm	31 mm
	Suitable annular dimensions			
Aortic annulus diameter (mm)	18–20	20–23	23–27	26–29
Aortic annulus area (mm^2^)	254–314	314–416	416–572	572–661
Aortic annulus perimeter (mm)	56–63	63–72	72–85	82–91

*mm* millimeter, *mm*^*2*^ square millimeter

### Statistics

Statistical analysis was conducted using Statistical Package for Social Sciences version 23.0 (SPSS Inc., Chicago, IL, USA). Continuous variables are expressed using descriptive statistics (mean value ± standard deviation [SD]). Categorical variables are expressed as frequencies and percentages. The mean values of aortic annulus dimensions were determined using a one-sample *t* test. Mean differences between aortic annulus diameters were calculated with Bland–Altman method with 95% limits of agreement (LOA; mean difference ± 1.96 × SD). Agreement between suggested prosthesis sizes is expressed as the percentage of patients in which the same valve size would be selected based on two or three different annular measurements. All p-values are based on a 2-sided α of 5%, and a p-value < 0.05 was considered statistically significant.

## Results

### Baseline characteristics

The baseline characteristics are summarized in Table 4. The study population consisted of 26 female and 24 male patients with an average age of 81 ± 5 years.

**Table 4:** Baseline characteristics, pre- and post-procedural aortic valve characteristics on echocardiography

	Mean ± SD	Range
Age (years)	81 ± 5	69–88
Height (cm)	167 ± 10	144–198
Weight (kg)	74 ± 12	52–109
Pre-procedural assessment		
Ejection fraction (%)	55 ± 12	24–73
Mean gradient (mmHg)	42 ± 17	11–77
Maximum gradient (mmHg)	69 ± 25	21–120
AVA (cm^2^)	0.82 ± 0.2	0.4–1.5
Post-procedural assessment (45)	5 ± 3 days after intervention	
Mean gradient (mmHg)	10 ± 4	4–22
Maximum gradient (mmHg)	20 ± 8	9–45
Aortic incompetence (AI)	1) Non/trace AI : 20 (44%) 2) Mild AI : 21 (47%) 3) Moderate AI: 4 (9%)	

*AI* aortic incompetence, *cm* centimeter, *cm*^*2*^ square centimeter, *mmHg* millimeter of mercury, *kg* kilogram, *SD* standard deviation, % percent

### Aortic annulus dimensions

The mean aortic annulus measurement was 22.3 ± 1.7 mm, 472.5 ± 63.8 mm^2^ and 79.3 ± 5.9 mm for short aortic annulus diameter, area and perimeter, respectively. The mean eccentricity of the aortic annulus was 0.58 ± 0.1 (minimum: 0.36; maximum: 0.73).

The summary of the mean annular diameter (short, long, mean, D_A_, D_P_) values and their mean difference from the LVOT in echocardiography are presented in Table 6 in Appendix 1.

### Transcatheter aortic valve prosthesis sizing

**Fig. 1:**
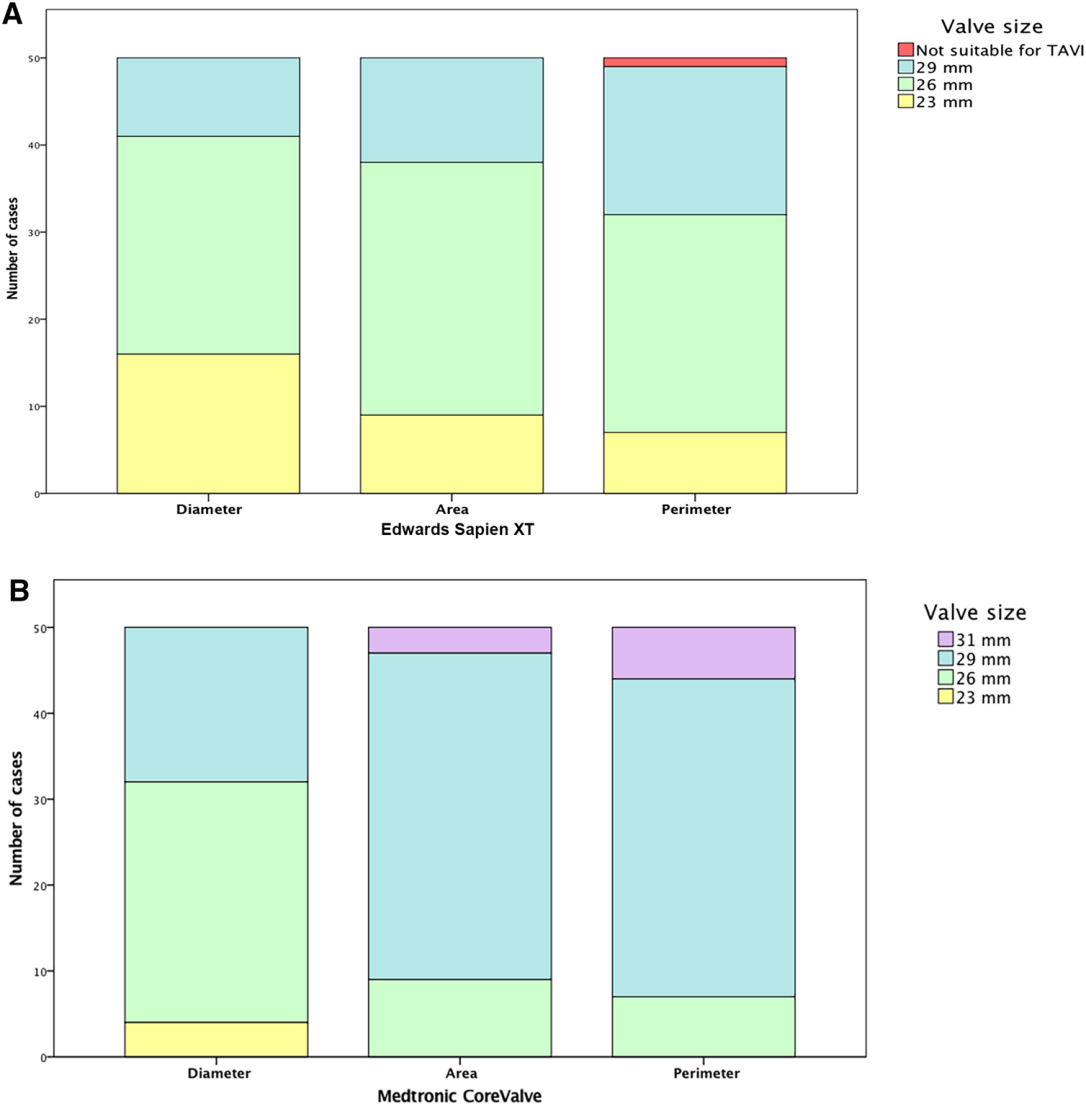
Bar graph of aortic valve size distribution derived from aortic annulus short diameter, area and perimeter. *mm* millimeter

Complete agreement on selected prosthesis size by all three annular dimensions was observed in 31 (62%; ESV) and 15 cases (30%; MCV). Short aortic annulus measurement resulted in a suggestion of one size smaller prosthesis size in 10 (20%; ESV) and in 30 cases (60%; MCV), compared to the size suggested by both annular perimeter and area. In 9 (18%; ESV) and 5 cases (10%; MCV), one size larger prosthesis would be selected based on annular perimeter compared to both annular diameter and area. Prosthesis size derived from the aortic annulus area was in agreement with at least one other parameter in all cases. A summary of suggested valve sizes derived from short annular diameter, area and perimeter is presented in Table 5 and Fig. 1.

**Table 5:**
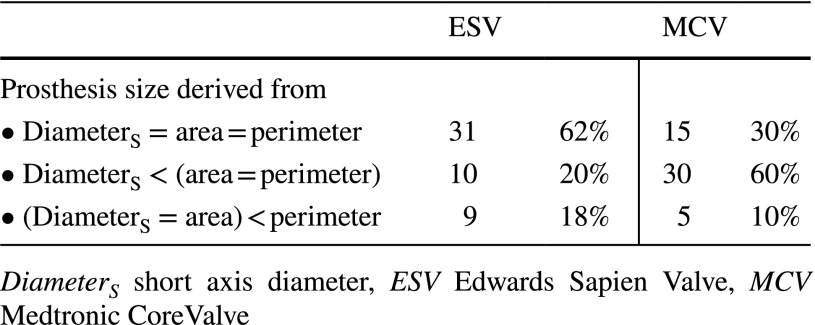
Comparison of transcatheter aortic valve sizes derived from short annular diameter, area and perimeter

Table 7 and Fig. 4 in Appendix 2 presents the comparison of valve sizes derived from annular diameters (short, long, mean, D_A_, D_P_).

## Discussion

This study clearly shows that annular diameter, area and perimeter dimensions are not interchangeable parameters in transcatheter aortic valve size selection, regardless of the prosthesis type. To the best of our knowledge, this is the first study that compares the differences in suggested prosthesis size between the three annular dimensions stated in the industry guidelines.

In this study, all three annular dimensions suggested the same prosthesis size in only 30–62% of cases. Based on these findings, 20–60% of the patients in this cohort could have been referred for a smaller valve size, perhaps leading to notable paravalvular regurgitation, device migration or embolization. In 10–18% of the study population, patients could be at risk of developing conduction disorders or even annular rupture, if referred for a larger than optimal valve [6, 15]. These results indicate that the matter of aortic valve sizing is considerably more complex than current guidelines would suggest.

**Fig. 2:**
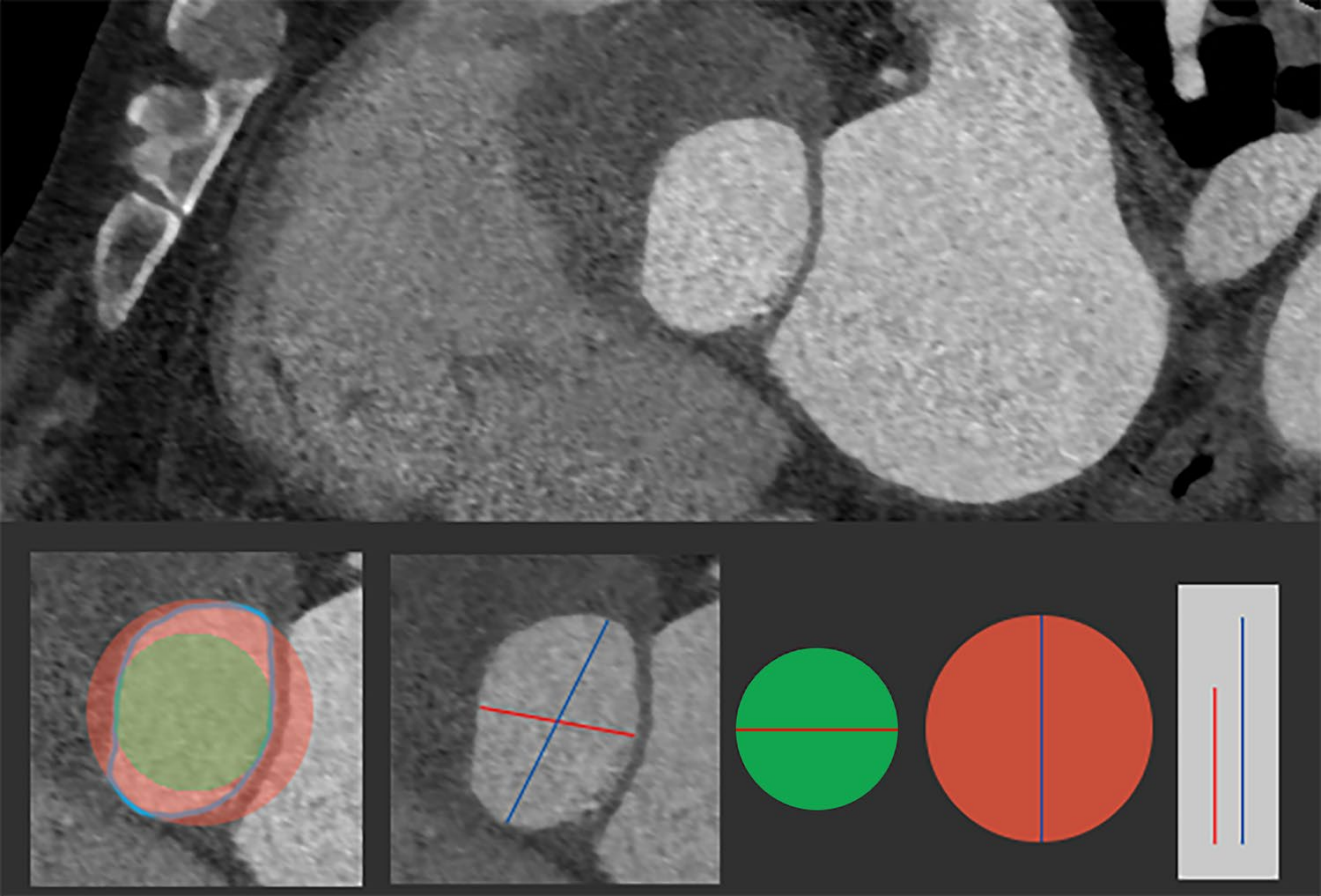
Use of one-dimensional diameters under the assumption of aortic annulus circularity may lead to underestimation (green circle; short diameter) or overestimation (red circle; long diameter) of optimal aortic valve prosthesis size. red line—short diameter; blue line—long diameter; green circular area—area of circle calculated from short diameter; red circular area—area of circle calculated from long diameter; light blue—outline/perimeter of native aortic annulus

The results of this study reveal that the transcatheter aortic valve sizing based on short annular diameter measurement has a tendency to underestimate the selected valve size, compared to a selection based on annular area and perimeter measurements. It is understandable that use of a one-dimensional parameter in prosthesis sizing might be prone to result in an unreliable estimation of prosthesis size, because it assumes circularity of the actually elliptical aortic annulus (Fig. 2). The use of three-dimensional annular dimension, and thus the use of MDCT should be therefore preferred over echocardiography in pre-procedural assessment [5–7]. Our results are in line with the findings of Schultz et al., who have reported the short diameter derived sizing to underestimate the prosthesis size in 50% of MCV cases when compared to direct surgical sizing [13]. This proportion of cases, potentially underestimating the prosthesis size, also corresponds with up to 67% incidence of paravalvular leak reported to follow the TAVI procedure [16, 17].

**Fig. 3:**
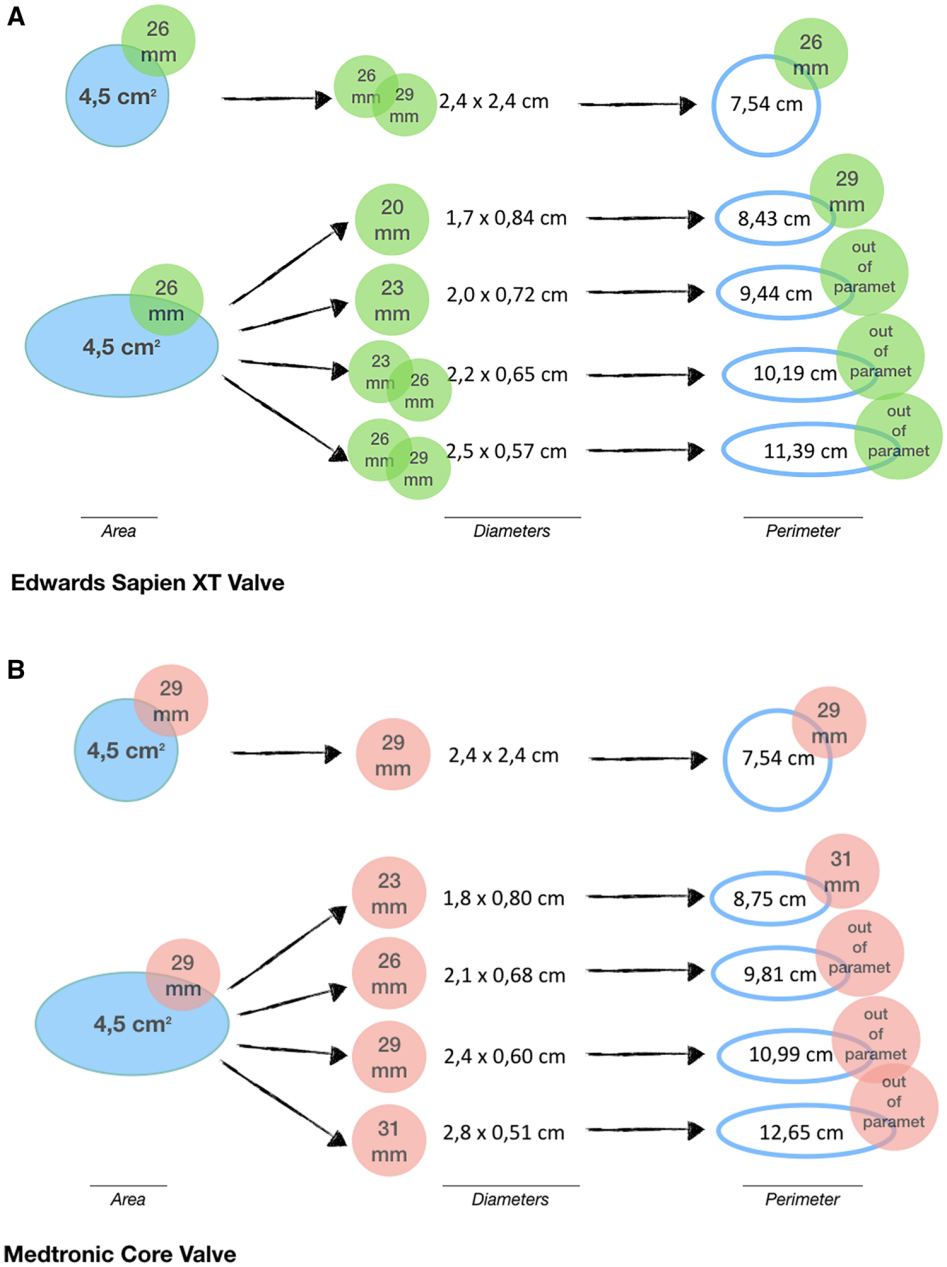
The area of the circle and all ellipses presented in this figure is constant (4.5 cm^2^). Figure showing how measure of perimeter increases with eccentricity of the elliptical aortic annulus and respective increase in the prosthesis valve size. full blue figure—circle or ellipse with an area of 4.5 cm^2^; blue outline—perimeter of circle or ellipse with different eccentricity, while the area of 4.5 cm^2^ is kept constant; green label—ESV valve size derived from the measurement (aortic annulus area, diameter or perimeter); red label—MCV valve size derived from the measurement (aortic annulus area, diameter or perimeter). *cm*^*2*^ square centimeter, *ESV* Edwards Sapien XT Valve, *MCV* Medtronic CoreValve, *mm* millimeter

**Fig. 4:**
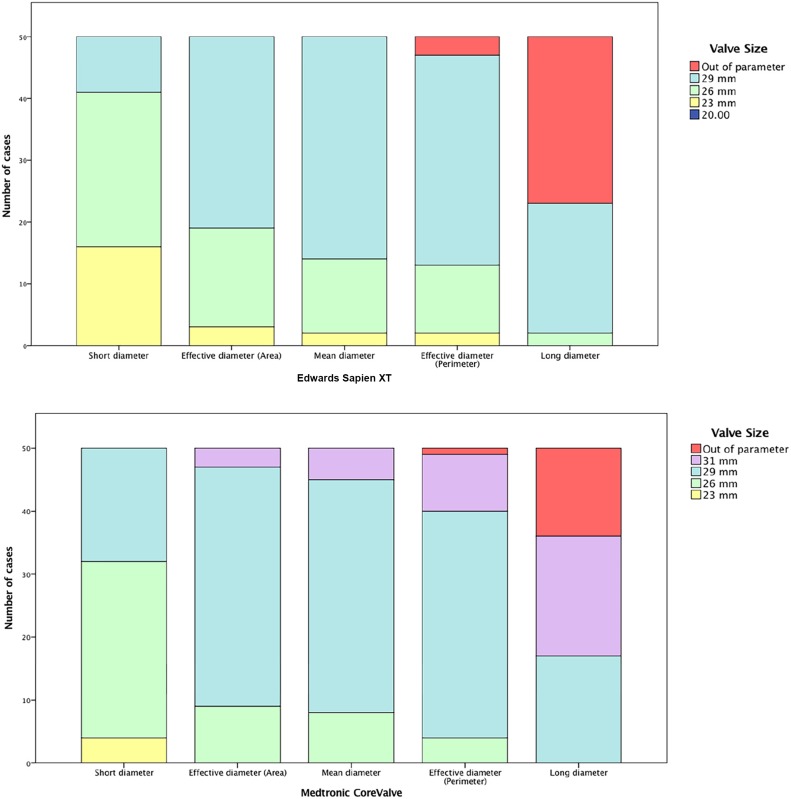
Transcatheter aortic valve size distribution derived from annular diameters (shown as number of cases). *D*_*A*_ area derived effective diameter, *D*_*P*_ perimeter derived effective diameter, *ESV* Edwards Sapien XT Valve, *MCV* Medtronic CoreValve, *mm* millimeter, not suitable—currently suitable valve size [ESV > 29 mm, MCV > 31 mm] not available

The results of this study also show that in some cases the measurement of annular perimeter has a tendency to result in larger valve size, compared to the consensual size derived from annular diameter and area. A combination of factors may be responsible for this overestimation. Manual assessment of the perimeter forms a rather polygonal than smooth annular silhouette [10]. Minimal irregularities and contour spikes can increase perimeter measurement leading to its overestimation and lower reproducibility, regardless of the post-processing software type [10, 15]. More importantly, the elliptical perimeter is proportionally amplified with increased eccentricity of the annular shape, in other words with greater difference between short and long diameter, even if the area of the ellipse remains the same (Fig. 3). This ellipse characteristic also complicates the use of effective diameters, which represent a diameter of an idealized circle with the same area or perimeter, regularly advised in literature for valve sizing [2, 11, 18]. When using effective diameters, it is important to consider the differences between devices, their characteristic in vivo shaping and their respective guideline set up. The MCV is a self-expandable valve, which adjusts to the elliptical annulus shape after deployment [3]. However, in the MCV guidelines the exact area and perimeter thresholds for each prosthesis size can be calculated with the dedicated formulas for a perfect circle from the annular diameter sizing thresholds. The general formulas for effective diameters are exactly reversed (see Table 2). From this, it follows that the absolute (100%) agreement for suggested MCV size between area and D_A_ is biased because they are mutually dependent, and the calculation of D_A_ therefore does not bring any additional value for MCV sizing. On the other hand, the ESV guidelines do not possess such a predictable relationship between diameter and area (or perimeter), even though the balloon-expandable ESV is likely to decrease annular eccentricity as it expands to circular shape in vivo [3, 12]. Thus, the ESV size derived from the annular area and D_A_ only agreed in 50% of cases. Also the perimeter and D_P_ would lead to selection of the same prosthesis size in only 48% and 84% of patients referred for ESV and MCV, respectively. It is not surprising, if the elliptical shape of the native aortic annulus is considered, that the use of D_P_ consistently leads to selection of a larger valve compared to short diameter (76–82%), D_A_ (20–24%), area (24–50%) and perimeter (16–54%; Table 8 in Appendix 3). Kim et al. have confirmed this overestimation also in comparison to the direct surgical sizing [19]. Based on our results, we believe that effective diameters should not be preferred over direct annular measurements, because they represent an indirect calculation, which assume a perfect annular circularity and therefore are prone to computational errors.

This study has demonstrated that the use of area-derived sizing thresholds in TAVI candidates results in consensus in prosthesis size with at least one other annular dimension in all patients, regardless on the valve type. The measurement of annular area appears to correspond the best with the industry recommended sizing thresholds because the shape of the native aortic annulus does not affect the measure of its area [20]. Together with reports of high inter- and intra-reader reproducibility [15], our results suggest that annular area is the most robust parameter in transcatheter aortic valve prosthesis size selection. Moreover, there might be additional benefits to the use of annular area in transcatheter aortic valve sizing. Willson et al. found the TAVI prosthesis sizing derived from the aortic annulus area to significantly reduce the risk of moderate or severe post-procedural paravalvular leakage (area under the curve: 0.80) [21]. Blanke et al. reported the annular area measurements on MDCT to be the best correlated parameter on pre- and post-TAVI datasets [12]. We therefore assume that eventual reshaping of aortic annulus after valve deployment is not likely to significantly influence annular area, therefore its consistency may allow for further and more sophisticated determination of necessary oversizing in order to minimize the risk of both paravalvular leak and annular injury.

### Limitations

This study is subject to some limitations. First of all, a relatively small population was evaluated. Although this study used annular measurements of actual TAVI candidates and thus mimics the common clinical decision-making process in transcatheter aortic valve prosthesis sizing, the results were established theoretically and should be viewed as hypothesis generating. Additionally, we have only evaluated the manufacturer recommendations for ESV and MCV devices, thus our results may not extrapolate to prosthetic valves of other manufacturers. This study assumed that after in vivo deployment the prosthesis would expand to its nominal size reported by the manufacturer. The retrospective character of this study does not permit confrontation of our results with clinical outcomes, procedural success or patient-prosthesis mismatch, because multiple transcatheter aortic devices were used during the evaluated period.

## Conclusion

Aortic annulus area appears to be the most robust parameter in transcatheter aortic valve prosthesis size selection. Short aortic annulus diameter may have a tendency to lead to selection of one size smaller prosthesis size, while use of annular perimeter may lead to selection of one size larger prosthesis size.

## Figures and Tables

**Table 7:**
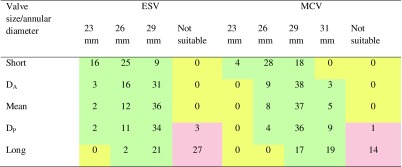
Transcatheter aortic valve size distribution derived from annular diameters (shown as number of cases)

*D*_*A*_ area derived effective diameter, *D*_*P*_ perimeter derived effective diameter, *ESV* Edwards Sapien XT Valve, *MCV* Medtronic CoreValve, *mm* millimeter, *not suitable* currently suitable valve size [ESV > 29 mm, MCV > 31 mm] not available

**Table 8:**
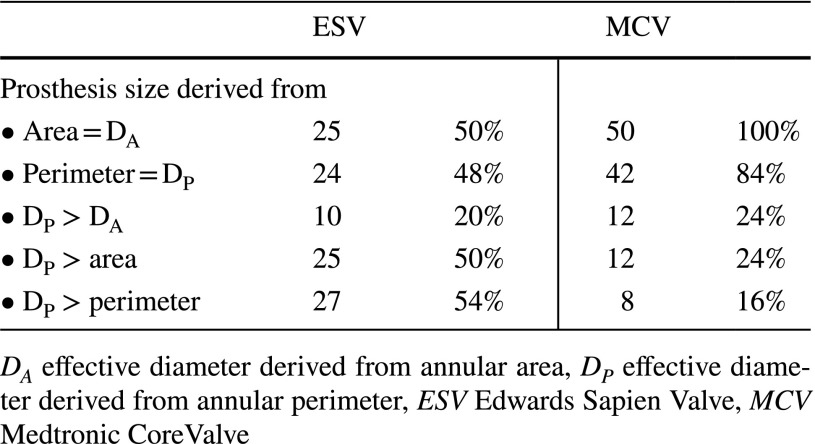
Comparison of transcatheter aortic valve sizes derived from annular effective diameters, area and perimeter (number and % of cases)

## Appendix 1

See Table 6.

**Table 6:** Mean annular dimensions derived from echocardiography (LVOT) and MDCT

Diameter	Mean ± SD [mm]	Mean difference from LVOT	95% LOA	Sign. (2-sided)
LVOT	21.1 ± 1.4			p < 0.001
Short	22.3 ± 1.7	1.3 ± 1.8	– 2.2 to 4.8	p < 0.001
D_A_	24.5 ± 1.7	3.4 ± 1.7	0.1 to 6.7	p < 0.001
Mean	25.0 ± 1.7	4.0 ± 1.7	0.7 to 7.3	p < 0.001
D_P_	25.2 ± 1.8	4.2 ± 1.8	0.7 to 7.3	p < 0.001
Long	27.7 ± 2.0	6.7 ± 2.0	2.8 to 10.6	p < 0.001

*D*_*A*_ area derived effective diameter, *D*_*p*_ perimeter derived effective diameter, *LOA* limits of agreement, *LVOT* left ventricular outflow tract, *MDCT* multidetector computed tomography, *mm* millimeter, *SD* standard deviation, *sign*. significance

## Appendix 2

See Table 7 and Fig. 4.

## Appendix 3

See Table 8.

## Abbreviations

CM: Contrast media
CTA: Computed tomographic angiography
D_A_: Effective diameter derived from aortic annulus area
D_P_: Effective diameter derived from aortic annulus perimeter
ESV: Edwards Sapien valve
IDR: Iodine delivery rate
IR: Iterative reconstruction
LVOT: Left ventricular outflow tract
MCV: Medtronic CoreValve
METC: Medical ethical research committee
MDCT: Multidetector row computed tomography
MPR: Multiplanar reformations
PLAX: Parasternal long axis view
ROI: Region of interest
SCCT: Society of cardiovascular computed tomography
SD: Standard deviation
SPSS: Statistical package for social sciences
TAVI: Transcatheter aortic valve implantation
